# Understanding Mental Health Issues in Different Subdomains of Social Networking Services: Computational Analysis of Text-Based Reddit Posts

**DOI:** 10.2196/49074

**Published:** 2023-11-30

**Authors:** Seoyun Kim, Junyeop Cha, Dongjae Kim, Eunil Park

**Affiliations:** 1 Department of Applied Artificial Intelligence Sungkyunkwan University Seoul Republic of Korea; 2 Teach Company Seoul Republic of Korea

**Keywords:** mental health, sentiment analysis, mental disorder, text analysis, NLP, natural language processing, clustering

## Abstract

**Background:**

Users increasingly use social networking services (SNSs) to share their feelings and emotions. For those with mental disorders, SNSs can also be used to seek advice on mental health issues. One available SNS is Reddit, in which users can freely discuss such matters on relevant health diagnostic subreddits.

**Objective:**

In this study, we analyzed the distinctive linguistic characteristics in users’ posts on specific mental disorder subreddits (depression, anxiety, bipolar disorder, borderline personality disorder, schizophrenia, autism, and mental health) and further validated their distinctiveness externally by comparing them with posts of subreddits not related to mental illness. We also confirmed that these differences in linguistic formulations can be learned through a machine learning process.

**Methods:**

Reddit posts uploaded by users were collected for our research. We used various statistical analysis methods in Linguistic Inquiry and Word Count (LIWC) software, including 1-way ANOVA and subsequent post hoc tests, to see sentiment differences in various lexical features within mental health–related subreddits and against unrelated ones. We also applied 3 supervised and unsupervised clustering methods for both cases after extracting textual features from posts on each subreddit using bidirectional encoder representations from transformers (BERT) to ensure that our data set is suitable for further machine learning or deep learning tasks.

**Results:**

We collected 3,133,509 posts of 919,722 Reddit users. The results using the data indicated that there are notable linguistic differences among the subreddits, consistent with the findings of prior research. The findings from LIWC analyses revealed that patients with each mental health issue show significantly different lexical and semantic patterns, such as word count or emotion, throughout their online social networking activities, with *P*<.001 for all cases. Furthermore, distinctive features of each subreddit group were successfully identified through supervised and unsupervised clustering methods, using the BERT embeddings extracted from textual posts. This distinctiveness was reflected in the Davies-Bouldin scores ranging from 0.222 to 0.397 and the silhouette scores ranging from 0.639 to 0.803 in the former case, with scores of 1.638 and 0.729, respectively, in the latter case.

**Conclusions:**

By taking a multifaceted approach, analyzing textual posts related to mental health issues using statistical, natural language processing, and machine learning techniques, our approach provides insights into aspects of recent lexical usage and information about the linguistic characteristics of patients with specific mental health issues, which can inform clinicians about patients’ mental health in diagnostic terms to aid online intervention. Our findings can further promote research areas involving linguistic analysis and machine learning approaches for patients with mental health issues by identifying and detecting mentally vulnerable groups of people online.

## Introduction

### Background

Recently, concerns have been raised about the rapid increase in the number of people with mental illness [1]. Moreover, according to the World Health Organization, the prevalence of patients with mental illness recently increased by 13% in a decade [2], and more than 264 million people were diagnosed with clinical depression in 2020. Because mental health problems, including depression, schizophrenia, bipolar disorder, autism, borderline personality disorder (BPD), and anxiety disorder, have significant impacts on individuals’ lives, they must be appropriately addressed [1,3-7]. Nevertheless, mental illnesses are rarely recognized or treated, which has various negative effects on society [8].

Against this background, as social networking services (SNSs) have become ubiquitous, research has shown that people freely express their opinions and feelings through them [9,10]. Moreover, people with mental disorders tend to use online SNSs to express their emotions and experiences [11], with users discussing their opinions and symptoms through online communities [12]. Consequently, SNSs have become valuable resources for analyzing their users’ mental disorders [8,13,14].

Among several well-known SNSs, *Reddit* is a topic-oriented service that allows users to exchange their opinions with other users about the same topics. This means that Reddit is an effective platform for investigating the linguistic characteristics of individuals with specific mental disorders by focusing on the Reddit subreddits for a particular disorder [3]. For instance, Reddit currently operates several subreddits for different mental disorders as follows: r/depression, r/anxiety, r/bipolar, r/BPD, r/schizophrenia, r/autism, and r/mentalhealth.

Moreover, Reddit is dedicated to providing a platform on which participants can discuss specific topics of interest. Users can write posts on what they want to discuss in the subreddit threads and respond to others’ posts by commenting. Various research groups have used Reddit subreddits to analyze the linguistic characteristics of particular communities. Yoo et al [15] used semantic network analysis using term frequency–inverse document frequency values. They showed that users active in both the r/bipolar and r/depression subreddits commonly discuss sleep disorder episodes and financial problems. They also found that users of the r/bipolar subreddit are more interested in medication and tend to express themselves using logical expressions. In contrast, users of the r/depression subreddit are more interested in suicide-related topics and tend to express themselves more using narrative terms. Pirina and Çöltekin [16] proposed an integrated data set for identifying depression among Reddit users and proposed a linear support vector machine with bag-of-n-gram features. They used a 5-fold cross-validation method and achieved an *F*_1_-score of 55.62%. In addition, Tadesse et al [17] conducted experiments to detect depression-related posts on Reddit and demonstrated that implementing fused Linguistic Inquiry and Word Count (LIWC), latent Dirichlet allocation (LDA), and bigram features is highly robust and effective for the task. They used a multilayer perceptron for their classification performance and achieved an accuracy of 91% and an *F*_1_-score of 93% in investigating the posts.

Numerous research groups have also investigated schizophrenia, a challenging mental illness to detect and differentiate due to its potential to be confused with other psychiatric disorders or the absence of specific biomarkers that aid in diagnosis [18,19]. Zomick et al [20] proposed a model for the automatic identification of users of the r/schizophrenia subreddit with LIWC features. They trained the logistic regression model with a 5-fold cross-validation approach, with the model achieving an accuracy of 81.56%.

Beyond analyzing individual communities with a single mental illness, some researchers have focused on structural differences between various communities. Given that the content on Reddit is divided into subreddits, researchers can easily collect and analyze posts on diverse mental health–related issues. Thus, many researchers have proposed classification models that can potentially classify, based on their posts, whether a specific user has a certain mental illness [21-24].

Although many studies on mental health have been performed using SNSs, most have mainly focused on a single mental illness community or solely on a classification approach. There is thus a need to investigate whether there are differentiable linguistic characteristics in users’ posts across multiple mental illness communities at the same level.

In this research, we attempted to present these differences among 7 subreddits on different mental disorders (r/depression, r/anxiety, r/bipolar, r/BPD, r/schizophrenia, r/autism, and r/mentalhealth) using a well-known sentiment analysis tools LIWC [25], along with ANOVA with a post hoc test. We identified several findings and the most discriminative features for each disorder presented in each subreddit based on statistical results. In addition to the characteristics of each unique subreddit, we endeavored to examine distinct user patterns across various subreddits involving multiple mental disorders through 3 kinds of clustering methods. Furthermore, we validated posts from these specific subreddits (mental health) externally by contrasting them with posts from general subreddits (non–mental health). This enabled us to ascertain whether posts of users with mental health conditions can be discerned based on their irregular communication patterns on SNSs.

### Objective

Throughout our research, we aimed to enhance our understanding of linguistic characteristics associated with various mental disorders and obtain valuable insights for early intervention strategies in individuals with these conditions. The insights gained from linguistic analysis can guide the development of data-driven mental health interventions, such as chatbots or personalized support systems, which are designed to respond to individuals’ linguistic cues and needs.

Moreover, by using a clustering approach to analyze the compared groups of posts, we demonstrated significant sentiment variations among these groups. We believe that this kind of analysis has the potential to enrich our comprehensive understanding of online mental health communities and can result in improved support, interventions, and mental health resources in the digital space.

Therefore, in short, we aimed to address the following research questions (RQs):

- RQ1: Can we identify distinct linguistic characteristics in users’ posts on SNSs that are associated with specific mental disorders through machine learning methods?
- RQ2: Can we capture variations in language patterns among individuals with multiple mental illnesses?
- RQ3: Can we capture the difference between mental health posts and non–mental health posts on SNSs?

## Methods

### Study Design

According to the 3 RQs, the experiments were divided into 3 main categories:

- Unisubreddit analysis: internal comparison among the different mental health subreddits to identify the distinctive characteristics of each mental disease
- Multisubreddit analysis: internal pairwise comparison of users who upload posts in more than 1 mental health subreddit to capture changes in language usage by subreddit
- External comparison: comparing posts of mental health subreddits with posts of non–mental health subreddits to recognize posts of users with mental illness out of SNS activities

Tables 1 and 2 show the overall summary of the experimental flow for the 3 tasks. Details of our experiments are provided later.

**Table 1:** Overall summary of the experimental flow for the 3 experimental tasks using sentiment analysis (LIWC^a^ feature).

Internal comparison			External comparison
Unisubreddit analysis	Multisubreddit analysis		
ANOVA	*t* Test	*t* Test	
Bonferroni post hoc test	N/A^b^	N/A	

^a^LIWC: Linguistic Inquiry and Word Count.

^b^N/A: not applicable.

**Table 2:** Overall summary of the experimental flow for the 3 experimental tasks using clustering (BERT^a^ embedding).

Internal comparison			External comparison
Unisubreddit analysis	Multisubreddit analysis		
Sentence-BERT clustering	N/A^b^	Sentence-BERT clustering	
Unsupervised clustering (k-means)	N/A	Unsupervised clustering (k-means)	
Supervised clustering (UMAP^c^)	N/A	Supervised clustering (UMAP)	

^a^BERT: bidirectional encoder representations from transformers.

^b^N/A: not applicable.

^c^UMAP: uniform manifold approximation and projection.

### Data

#### Data Collection

To determine the linguistic characteristics of users with each mental disorder and compare r/mentaldisorder subreddit posts with other general subreddit posts, we collected both mental health and non–mental health data.

For mental health data, we first collected posts from 6 subreddits (r/depression, r/anxiety, r/bipolar, r/BPD, r/schizophrenia, and r/autism), which were mainly investigated and confirmed in previous research to be the mental health–related subreddits that were most active using both statistical and expert assessment approaches [26]. We also collected users’ posts in r/mentalhealth, one of the most popular mental health–related subreddits [27].

For non–mental health data, we collected posts from 4 subreddits discussing general topics (r/cleanjokes, r/conspiracy, r/relationships, and r/teaching), which were selected referring to the findings of previous studies that attempted to compare mental health and non–mental health groups on SNSs [3,28].

Compared to previous research, which focused on users’ posts on Reddit in only a limited period [22], we aimed to use more extended data sets (January 2018-December 2022) that are more reliable for capturing the recent trends and changes occurring in SNSs.

All user IDs, titles, and posts from each subreddit were collected using the Pushshift application programming interface. We collected 1,303,985 posts of 419,426 users on the 7 mental health subreddits, while collecting 1,829,524 posts of 500,296 users on the 4 non–mental health subreddits. An overall summary of our final data set, including the numbers of users and submissions, is presented in Table 3.

**Table 3:** Overall summary of the collected data.

Subreddit category and types			Users, n/N (%)		Posts, n/N (%)
**Mental health subreddits**					
	r/depression	160,571/419,426 (38.3)		515,674/1,303,985 (39.5)	
	r/anxiety	74,560/419,426 (17.8)		210,274/1,303,985 (16.1)	
	r/mentalhealth	79,960/419,426 (19.1)		212,255/1,303,985 (16.3)	
	r/BPD^a^	33,106/419,426 (7.9)		127,961/1,303,985 (9.8)	
	r/bipolar	30,479/419,426 (7.3)		112,846/1,303,985 (8.7)	
	r/autism	31,326/419,426 (7.5)		90,201/1,303,985 (6.9)	
	r/schizophrenia	9424/419,426 (2.3)		34,774/1,303,985 (2.7)	
**Non–mental health subreddits**					
	r/relationships	394,861/500,296 (78.9)		1,310,074/1,829,524 (71.6)	
	r/conspiracy	91,853/500,296 (18.4)		486,289/1,829,524 (26.6)	
	r/teaching	11,685/500,296 (2.3)		24,112/1,829,524 (1.3)	
	r/cleanjokes	1897/500,296 (0.4)		9049/1,829,524 (0.5)	

^a^BPD: borderline personality disorder.

#### Data Processing

We conducted a series of data preprocessing procedures that have been validated in previous studies [22]. Initially, relevant data elements (eg, author, title, text, and subreddit information) were extracted from the entire data set. Subsequently, samples with deleted accounts were removed, along with any text information that was either empty or deleted.

**Figure 1:**
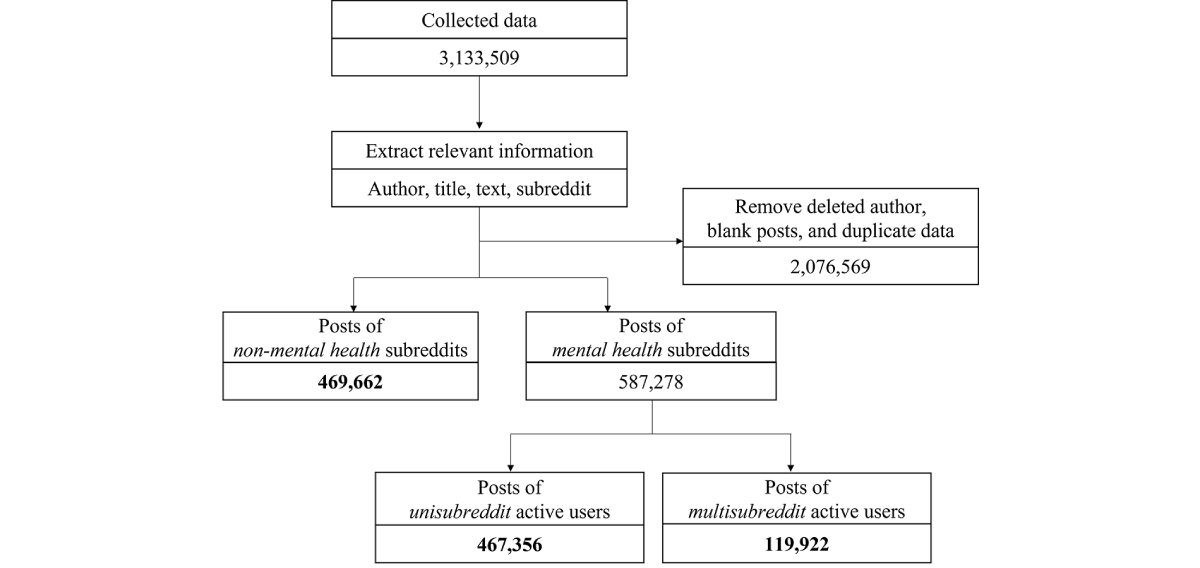
A flowchart of the preprocessing of Reddit posts.

For mental health data, we implemented a user-centric filtering approach because users can be active in multiple subreddits, uploading textual posts simultaneously in multiple subreddits. Considering that the primary objective of the unisubreddit analysis was to discern the unique linguistic attributes of users with each mental disorder, we selectively filtered the data of users who were only active in a specific subreddit (ie, unisubreddit data), sparing those who engaged in multiple subreddits (ie, multisubreddit data) for further multisubreddit analysis. Figure 1 shows all data processing procedures, while Tables 4 and 5 summarize the amount of data in each process for mental health and non–mental health data, respectively.

**Table 4:** Changes in the amount of mental health data throughout data processing.

Subreddit	Original data (N=1,303,985), n (%)	Preprocessed data (N=587,278), n (%)	Unisubreddit data (N=467,356), n (%)	Multisubreddit data (N=119,922), n (%)
r/depression	515,674 (39.5)	225,854 (38.5)	187,487 (40.1)	38,367 (32.0)
r/anxiety	210,274 (16.1)	103,948 (17.7)	79,755 (17.1)	25,751 (21.5)
r/mentalhealth	212,255 (16.3)	101,383 (17.3)	78,197 (16.7)	21,628 (18.0)
r/BPD^a^	127,961 (9.8)	55,365 (9.4)	42,968 (9.2)	12,397 (10.3)
r/bipolar	112,846 (8.7)	48,462 (8.3)	37,256 (8.0)	11,206 (9.3)
r/autism	90,201 (6.9)	37,896 (6.5)	31,495 (6.7)	6401 (5.3)
r/schizophrenia	34,774 (2.7)	14,370 (2.5)	10,198 (2.2)	4172 (3.5)

^a^BPD: borderline personality disorder.

**Table 5:** Changes in the amount of non–mental health data throughout data processing.

Subreddit	Original data (N=1,829,524), n (%)	Preprocessed data (N=469,662), n (%)
r/relationships	1,310,074 (71.6)	240,706 (51.3)
r/conspiracy	486,289 (26.6)	210,100 (44.7)
r/teaching	24,112 (1.3)	11,600 (2.5)
r/cleanjokes	9049 (0.5)	7256 (1.5)

### Sentiment Analysis

We used LIWC software to conduct sentiment analysis of posts published by users of Reddit subreddits related to mental health. LIWC is a text-mining tool that classifies words into categories, including grammatical, psychological (affective, perceptual, cognitive, etc), and personal (occupation, leisure activity, etc) content [25]. The software calculates several sentiment scores based on the frequency of the appearance of these words in the given text. Specifically, LIWC identifies positive or negative terms, as well as social words and other relevant categories of interest. LIWC has been widely adopted as a tool for analyzing the emotional content of texts in various research domains, as demonstrated in previous research [3,11,14,20,29].

To determine the linguistic differences among the distinct 7 mental health subreddits (unisubreddit analysis), ANOVA with Bonferroni post hoc tests was sequentially implemented on the features driven by LIWC analysis in order to find out whether the differences between different groups of data are statistically significant.

Another objective was to determine whether the users who are active in multiple mental health subreddits show different linguistic patterns in each subreddit (multisubreddit analysis). Within the data of the same users posting in different subreddits, we conducted pairwise independent *t* tests on the LIWC features.

Likewise, for external comparison, where we aimed to determine whether we could differentiate the linguistic features in posts of users with mental issues from those of general SNS users, an independent *t* test was conducted between the 2 groups of posts.

We measured the effect sizes (Cohen d [30] and Cohen f [30]) for the results of the *t* test and ANOVA, respectively, to provide the actual clinical significance.

### Clustering

In addition to the statistical approaches mentioned before, we extracted features for the users’ posts using a large-scale language model. The objective was to confirm whether the linguistic differences among individuals with mental illness can be captured with machine learning techniques. We applied clustering methods to show the distinctiveness internally (among the mental health posts of different subreddits; unisubreddit analysis) and externally (between non–mental health and mental health posts; external comparison). Through them, we determined whether there were any tendencies or trends, such as overlap or distinctiveness, among the groups. However, the r/mentalhealth subreddit was excluded from unisubreddit analysis because we assumed that the r/mentalhealth subreddit acts as a central hub where all users with the 6 mental health problems coexist, conferring it with a high level of generality.

For unisubreddit analysis (6 groups) and external comparison (2 groups), the following steps were taken:

- We applied clustering approaches to the sentence-BERT-based [31] vector representation of our collected discourses. We fed the post text as input into the model to obtain a 384-dimensional embedding vector for each text input.
- We analyzed the extracted embeddings using 3 types of clustering methods: the clustering method inside the sentence-BERT package [31], k-means clustering, and uniform manifold approximation and projection (UMAP).
- We applied the community detection method inside the sentence-BERT to check whether the extracted feature vectors could be clustered well following its subreddit. Through this approach, we identified whether further clustering approaches were practical for data analysis. For this analytical step, we used a threshold of 0.75 for cosine similarity.
- Next, we applied k-means algorithms to ensure that the embedding vectors could distinguish among the groups without supervision. For unisubreddit analysis, we used subsequent unsupervised k-means clustering with randomly undersampled embedding vectors to handle data imbalance.
- We used the scikit-learn function to determine clusters with default parameters and an array of 6 initial centroids. We reduced the 384-dimensional feature vectors to 2D using t-distributed stochastic neighbor embedding (t-SNE) for visualization after clustering.
- Finally, we performed the hierarchical density-based spatial clustering of applications with noise (HDBSCAN) algorithm to confirm that the sentence-BERT embedding vectors in each cluster could be differentiated from the others. By applying this approach, we checked whether each group shows distinctive properties that can be used to distinguish it from the others.

For this approach, we performed 1-vs-rest supervised projection using the UMAP algorithm for each subreddit in unisubreddit analysis. In this context, we labeled the data as positive if they belonged to each subreddit and negative otherwise. Next, we applied HDBSCAN on the projected vectors to classify them into 2 groups. For external comparison, the same procedure was performed between the non–mental health and mental health groups. We also reported quantitative metrics to prove that such a classification is effective.

### Ethical Considerations

The purpose of using Reddit is to share opinions, information, or content. Therefore, users are generally aware that their data are publicly available within the terms of the Reddit site. We collected all the data from subreddits that are freely accessible to anyone, and we anonymized all personal identifying information, such as user ID and profile images. Since we did not engage in the subreddit discussions, there was no ethical requirement to alert users that their posts would be used for research. Furthermore, this study was approved by the Ethical Committee and Institutional Review Board of Sungkyunkwan University (no. 2020-11-025).

## Results

### Sentiment Analysis

#### Unisubreddit Analysis

**Figure 2:**
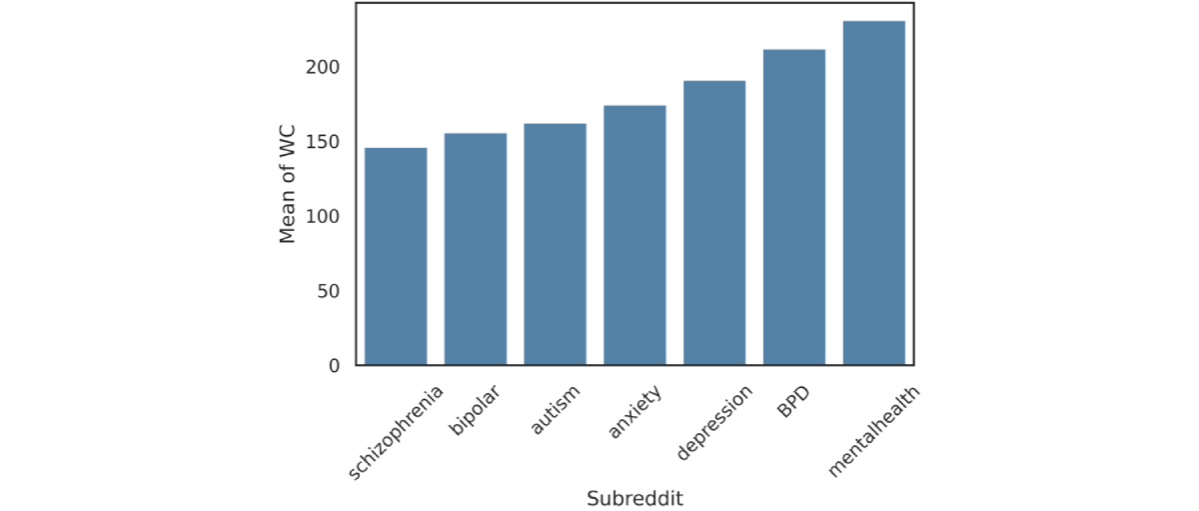
Average word count (WC) of each channel.

ANOVA and subsequent post hoc tests were performed to identify variations in sentiment analyses across the 7 subreddit groups. The results showed that the number of words per sentence of the posts in r/mentalhealth (mean 230.86, SD 260.2) was higher than those in other subreddits, followed by r/depression (mean 190.78, SD 224.46) and r/anxiety (mean 174.31, SD 177.15; *F*_6, 467,356_=920.72, *P*<.001, f=0.13; Figure 2). We also found that users in r/mentalhealth (mean 0.52, SD 1.04) tended to use more family-related words than users in the other subreddits (*F*_6, 467,356_=943.31, *P*<.001, f=0.11).

Posts in r/anxiety tended to include a higher number of terms associated with affective process (mean 8.13, SD 3.82; *F*_6, 467,356_=3421.54, *P*<.001, f=0.25), negative emotion (mean 5.69, SD 3.46; *F*_6,467,356_=57,838.29, *P*<.001, f=0.31), and anxiety (mean 2.97, SD 2.36; *F*_6, 467,356_=25,387.00, *P*<.001, f=0.46) than posts in the other subreddits. Furthermore, they presented more body-related words (eg, cheek and hand; mean 1.42, SD 2.19; *F*_6, 467,356_=2828.35, *P*<.001, f=0.18). They also tended to present more expressions related to power (mean 2.29, SD 1.90; *F*_6, 467,356_=535.08, *P*<.001, f=0.09), risk (mean 0.91, SD 1.18; *F*_6, 467,356_=349.86, *P*<.001, f=0.08), and time (mean 6.78, SD 3.51; *F*_6, 467,356_=1860.99, *P*<.001, f=0.20).

Users in r/BPD were found to use more third-person singular words (mean 1.64, SD 2.52; *F*_6, 467,356_=1843.80, *P*<.001, f=0.17) and first-person plural words in their posts (eg, we, us, our; mean 0.43, SD 0.91; *F*_6, 467,356_=526.90, *P*<.001, f=0.09). In addition, they used more terms related to friends (mean 0.61, SD 0.95; *F*_6, 467,356_=764.00, *P*<.001, f=0.11), affiliations (mean 2.31, SD 2.14; *F*_6, 467,356_=1192.86, *P*<.001, f=0.15), and female references (mean 0.83, SD 1.93; *F*_6, 467,356_=615.79, *P*<.001, f=0.10).

**Figure 3:**
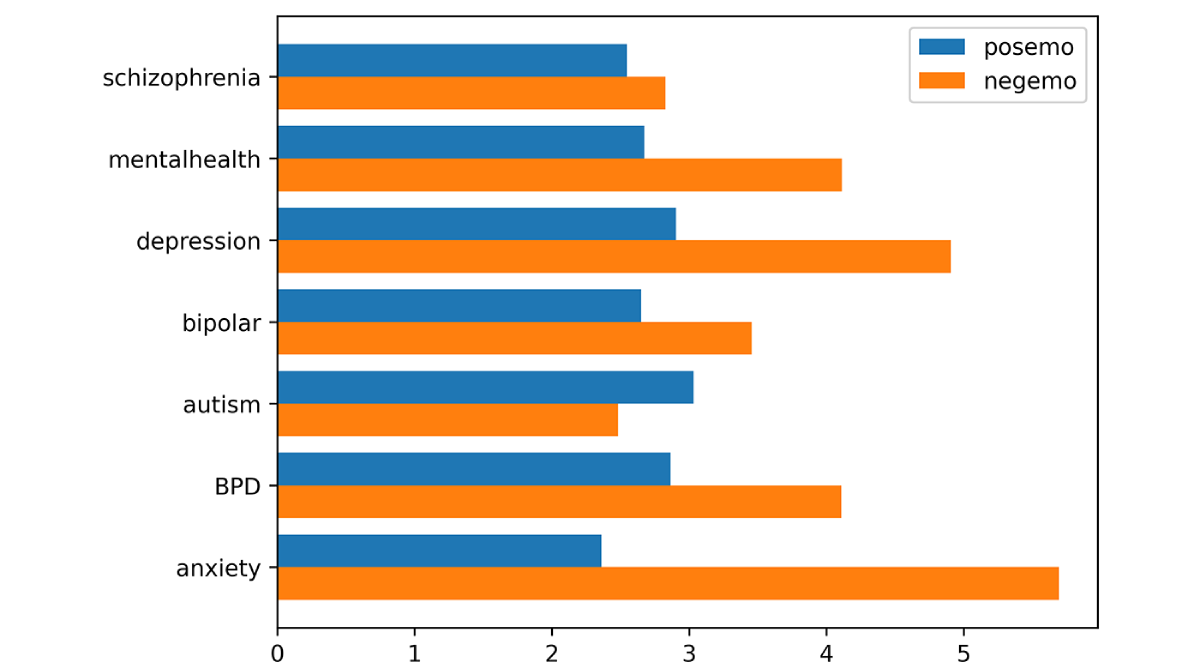
Results of sentiment analysis (positive and negative emotional aspects). negemo: negative emotion; posemo: positive emotion.

In r/autism, users’ posts tended to convey higher levels of confidence (eg, clout; mean 38.19, SD 29.36; *F*_6, 467,356_=3666.76, *P*<.001, f=0.25) and emotional tone (mean 40.22, SD 34.43) than posts in other subreddits (*F*_6, 467,356_=3948.41, *P*<.001, f=0.27). They tended to contain more positive (mean 3.03, SD 2.92; *F*_6, 467,356_=599.28, *P*<.001, f=0.09; Figure 3) and tentative (mean 4.63, SD 3.01; *F*_6, 467,356_=1056.46, *P*<.001, f=0.12) words, and more netspeak words were included in the posts (mean 0.72, SD 1.98; *F*_6, 467,356_=409.27, *P*<.001, f=0.09).

Users’ posts in r/bipolar tended to show higher frequencies of words showing nonfluency (eg, hm, umm, er; mean 0.15, SD 0.55; *F*_6, 467,356_=52.51, *P*<.001, f=0.03) and number-related terms (mean 1.41, SD 1.75; *F*_6, 467,356_=334.89, *P*<.001, f=0.08). They were found to contain words relevant to ingestion (eg, dish, eat, pizza; mean 0.51, SD 1.50; *F*_6, 467,356_=311.24, *P*<.001, f=0.07) and reward (eg, benefit, prize; mean 1.77, SD 1.86; *F*_6, 467,356_=436.74, *P*<.001, f=0.09), while reflecting more personal concerns regarding money (mean 0.35, SD 1.08; *F*_6, 467,356_=167.91, *P*<.001, f=0.05) than in other subreddits.

The r/depression subreddit posts were distinctive in that they presented greater numbers of first-person singular (mean 12.18, SD 4.62; *F*_6, 467,356_=4982.71, *P*<.001, f=0.26) and negation (mean 3.31, SD 2.30; *F*_6, 467,356_=4732.03, *P*<.001, f=0.20) terms. They also showed higher discrepancy (mean 2.29, SD 2.14; *F*_6, 467,356_=932.96, *P*<.001, f=0.09) with the abundant use of vocabulary such as “should” and “would” and the expression of certainty (mean 2.15, SD 1.92; *F*_6, 467,356_=1666.65, *P*<.001, f=0.13) using words such as “always” and “never.” Furthermore, such posts had a propensity to contain more words implying emotions of sadness (mean 1.88, SD 2.14; *F*_6, 467,356_=7566.71, *P*<.001, f=0.25) and anger (mean 1.28, SD 2.18; *F*_6, 467,356_=1504.27, *P*<.001, f=0.13). They also contained more vocabulary associated with the perception of feeling (eg, feels, touch; mean 1.68, SD 1.92; *F*_6, 467,356_=1049.94, *P*<.001, f=0.14). Sexual terms (mean 0.34, SD 1.28; *F*_6, 467,356_=713.51, *P*<.001, f=0.08) and terms related to achievement (eg, win, success, better; mean 1.64, SD 1.72; *F*_6, 467,356_=493.08, *P*<.001, f=0.08) were also more common. Moreover, users were more inclined to use present-focused expressions (mean 15.98, SD 5.21; *F*_6, 467,356_=2626.96, *P*<.001, f=0.14) than in other subreddits, while using more terms related to personal concerns regarding home (mean 0.41, SD 0.83; *F*_6, 467,356_=416.98, *P*<.001, f=0.07) and death (mean 0.58, SD 1.35; *F*_6, 467,356_=2839.51, *P*<.001, f=0.15).

Posts in r/schizophrenia showed a tendency to contain more words referring to the second person (eg, you; mean 1.49, SD 2.86; *F*_6, 467,356_=524.94, *P*<.001, f=0.12). They also presented more perceptual process-related terminology (mean 3.79, SD 3.61; *F*_6, 467,356_=360.63, *P*<.001, f=0.14) and words in the category of hearing (mean 1.21, SD 2.39; *F*_6, 467,356_=1324.48, *P*<.001, f=0.26). Lastly, posts in the schizophrenia subreddit tended to contain more words associated with personal concerns about religion (mean 0.27, SD 1.19; *F*_6, 467,356_=249.94, *P*<.001, f=0.12).

Table 6 summarizes the mean of the variables for each subreddit. These variables confirmed that differences between all group pairs of means were significant. For all statistical details of significant variables with more information, see Multimedia Appendix 1.

**Table 6:** Significant variables after ANOVA and post hoc tests for mental health–related subreddits.

Variable	r/anxiety, mean (SD)	r/BPD^a^, mean (SD)	r/autism, mean (SD)	r/bipolar, mean (SD)	r/depression, mean (SD)	r/mentalhealth, mean (SD)	r/schizophrenia, mean (SD)	*F*_6, 467,356_	*P* value	Effect size (f)
Wordcount	174.31 (177.15)	211.74 (220.91)	162.19 (179.66)	155.68 (177.72)	190.78 (224.46)	230.86^b^ (260.2)	145.93^c^ (205.61)	920.72	<.001	0.13 (very small)
Clout	21.84 (22.58)	27.91 (24.8)	38.19^b^ (29.36)	26.09 (25.8)	19.05^c^ (23.09)	25.49 (27.07)	34.98 (30.31)	3666.76	<.001	0.25 (small-medium)
Tone	13.41^c^ (23.19)	25.13 (29.97)	40.22^b^ (34.43)	28.67 (31.3)	21.31 (28.37)	23.09 (29.01)	32.92 (32.81)	3948.41	<.001	0.27 (small-medium)
Adverb	6.79 (2.96)	7.13 (2.75)	6.36 (3.24)	6.5 (3.17)	7.37^b^ (3.41)	6.72 (2.99)	6.02^c^ (3.49)	1128.52	<.001	0.13 (very small)
Affect	8.13^b^ (3.82)	7.16 (3.16)	5.61 (3.57)	6.32 (3.72)	7.91 (4.07)	6.92 (3.45)	5.47^c^ (3.83)	3421.54	<.001	0.25 (small-medium)
Anger	0.84 (1.46)	1.13 (1.58)	0.57^c^ (1.25)	0.76 (1.5)	1.28^b^ (2.18)	0.87 (1.43)	0.62 (1.37)	1504.27	<.001	0.13 (very small)
Sad	0.68 (1.09)	1.05 (1.31	0.45^c^ (0.93)	1.01 (1.6)	1.88^b^ (2.14)	1.11 (1.47)	0.56 (1.22)	7566.71	<.001	0.25 (small-medium)
Social	6.49^c^ (4.72)	9.7 (5.1)	9.84^b^ (5.54)	6.94 (5.09)	7.49 (5.25)	8.21 (5.43)	8.78 (6.25)	2979.56	<.001	0.23 (small)
Affiliation	1.46 (1.89)	2.31^b^ (2.14)	2.08 (2.38)	1.4^c^ (1.91	1.83 (2.12	1.99 (2.19)	1.62 (2.47)	1192.86	<.001	0.15 (very small)
Work	1.61 (2.13)	1.06^c^ (1.53)	1.86^b^ (2.49)	1.5 (2.1)	1.43 (1.91)	1.55 (1.97)	1.36 (2.22)	608.93	<.001	0.11 (very small)

^a^BPD: borderline personality disorder.

^b^Highest value.

^c^Lowest value.

#### Multisubreddit Analysis

**Figure 4:**
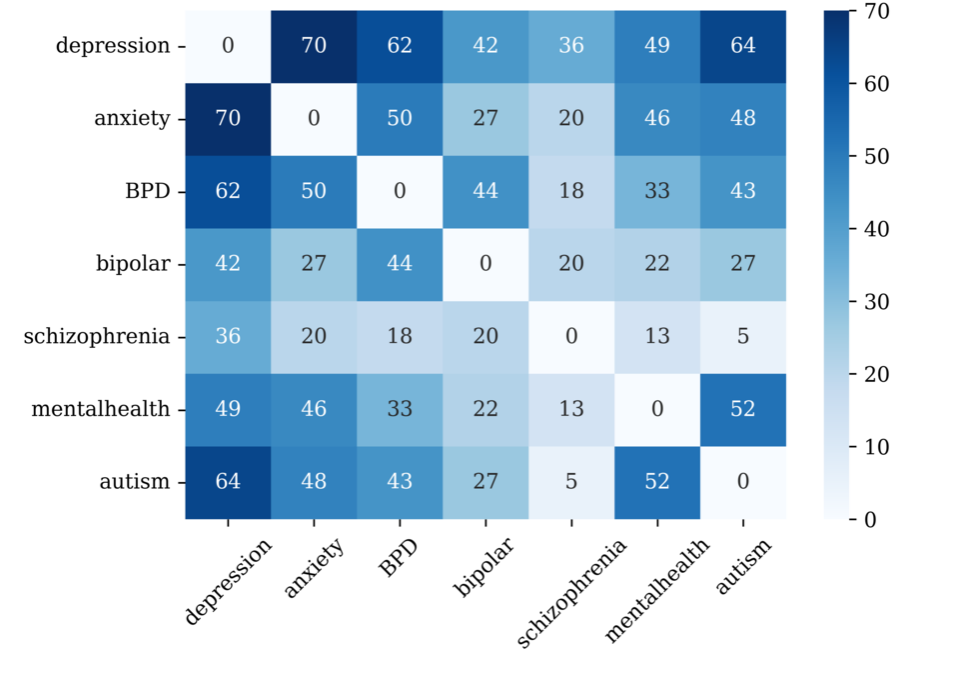
Results of pairwise *t* test for multisubreddit data. Each number in the heatmap represents the number of variables whose mean difference is statistically significant. BPD: borderline personality disorder.

A series of paired *t* tests was performed for every LIWC variable to identify variations in sentiment analysis between 2 subreddit groups in a pairwise comparison for the same users who were active in multiple subreddits. Figure 4 summarizes the number of significant variables resulting from the sequential *t* test on each subreddit pair.

Overall, the result revealed that multisubreddit users tended to show different tendencies in their language use on SNSs. The number of variables with a significant mean difference varied from 5 to 70. Specifically, we found that the posts of users who were simultaneously active in both r/anxiety and r/depression showed the difference in the maximum LIWC variables (n=70), followed by users active in both r/autism and r/depression (n=64) and in both r/BPD and r/depression (n=62). Meanwhile, posts of users active in both r/schizophrenia and r/autism showed the least difference in linguistic features (n=5).

Table 7 describes the statistics of variables with the largest effect size (Cohen d) in each pair. This represents the most pronounced language usage difference between the 2 subreddits of the same users who were active simultaneously in both.

**Table 7:** Statistics of variables with the largest effect size in each subreddit pair.

Subreddit and its comparison with other subreddits		Variable	*t* (*df*)	*P* value	Effect size (d)
**r/depression**					
	vs r/anxiety	Number	–3.49 (40,070)	<.001	0.0005 (very small)
	vs r/mentalhealth	Article	–3.30 (25,943)	<.001	0.0010 (very small)
	vs r/bipolar	Swear	3.34 (8044)	<.001	0.0008 (very small)
	vs r/BPD^a^	You	–3.30 (9864)	<.001	0.0010 (very small)
	vs r/autism	Differ	–3.41 (5010)	<.001	0.0007 (very small)
	vs r/schizophrenia	Focusfuture	3.51 (2254)	<.001	0.0005 (very small)
**r/anxiety**					
	vs r/mentalhealth	Verb	–3.43 (17,461)	<.001	0.0006 (very small)
	vs r/bipolar	Cogproc	–3.45 (5361)	<.001	0.0006 (very small)
	vs r/BPD	Swear	–3.33 (5079)	<.001	0.0009 (very small)
	vs r/autism	Space	3.41 (3707)	<.001	0.0007 (very small)
	vs r/schizophrenia	Article	–3.37 (1436)	<.001	0.0008 (very small)
**r/mentalhealth**					
	vs r/bipolar	Negemo^b^	3.30 (5486)	<.001	0.0010 (very small)
	vs r/BPD	Feel	–3.52 (6658)	<.001	0.0004 (very small)
	vs r/autism	Affiliation	–3.39 (4895)	<.001	0.0007 (very small)
	vs r/schizophrenia	Adj	3.32 (2707)	<.001	0.0009 (very small)
**r/bipolar**					
	vs r/BPD	Clout	–3.45 (5140)	<.001	0.0006 (very small)
	vs r/autism	Death	3.41 (1691)	<.001	0.0007 (very small)
	vs r/schizophrenia	Dic	–3.43 (2439)	<.001	0.0006 (very small)
**r/BPD**					
	vs r/autism	Motion	3.31 (2149)	<.001	0.0010 (very small)
	vs r/schizophrenia	Anger	3.35 (940)	<.001	0.0009 (very small)
**r/autism**					
	vs r/schizophrenia	Health	–3.76 (985)	<.001	0.0002 (very small)

^a^BPD: borderline personality disorder.

^b^Negemo: negative emotion.

Although the effect sizes were interpreted to be small, the majority of variables means differed significantly between the 2 groups, as shown in Figure 4. For more statistical details of the significant variables of each pair, see Multimedia Appendix 1.

#### External Comparison

For the external comparison, where we compared non–mental health and mental health data, an independent *t* test was implemented for each LIWC variable. As a result, 90 (96.8%) of 93 variables were shown to have significant mean differences. Table 8 summarizes the statistical details of the top 10 variables for each subreddit sorted by effect size.

**Table 8:** Statistical details of top 10 significant variables with effect size (Cohen d).

Variable	Non–mental health group, mean (SD)	Mental health group, mean (SD)	*t* (*df*)	*P* value	Effect size (d)
I	5.67 (4.30)	11.04 (4.55)	586.86 (937,016)	<.001	1.21 (very large–huge)
Authentic	49.34 (29.36)	80.48 (21.97)	523.38 (937,016)	<.001	1.08 (large–very large)
Negemo^a^	2.32 (1.90)	4.50 (3.34)	388.92 (937,016)	<.001	0.80 (large–very large)
Focuspresent	11.45 (4.34)	14.93 (4.97)	361.19 (937,016)	<.001	0.75 (medium-large)
Affect	4.95 (2.73)	7.37 (1.73)	350.14 (937,016)	<.001	0.72 (medium-large)
Dic	86.04 (12.17)	92.79 (5.78)	342.63 (937,016)	<.001	0.71 (medium-large)
Sad	0.42 (0.71)	1.28 (1.77)	307.31 (937,016)	<.001	0.63 (medium-large)
Verb	17.65 (5.11)	20.70 (4.69)	300.29 (937,016)	<.001	0.62 (medium-large)
Feel	0.65 (0.90)	1.53 (1.81)	299.15 (937,016)	<.001	0.62 (medium-large)
Health	0.77 (1.20)	1.78 (2.17)	280.59 (937,016)	<.001	0.58 (medium-large)

^a^Negemo: negative emotion.

In addition to the fact that almost all the features differed from one another, detailed descriptions of specific variables are as follows.

The mental health group (mean 11.04, SD 4.55) showed a higher frequency in the use of first-person singular terms than the non–mental health group (mean 5.67, SD 4.30), showing the largest effect size of 1.21. Moreover, users of mental health subreddits (mean 80.48, SD 26.23) tended to show a higher authenticity than users of general subreddits (mean 49.34, SD 31.13), with an effect size of 1.08, which is considered large. In addition, the mental health group showed a tendency of using more overall affective terms (mean 7.37, SD 3.87), along with words related to negative emotion (mean 4.50, SD 3.34) and sadness (mean 1.28, SD 1.77) compared to the non–mental health group. Posts of the mental health group also contained more vocabulary associated with the perception of feeling (mean 1.53, SD 1.81) and health-related terms (mean 1.78, SD 2.17). For all statistical details of significant variables, see Multimedia Appendix 1.

### Clustering

#### Internal Comparison

**Figure 5:**
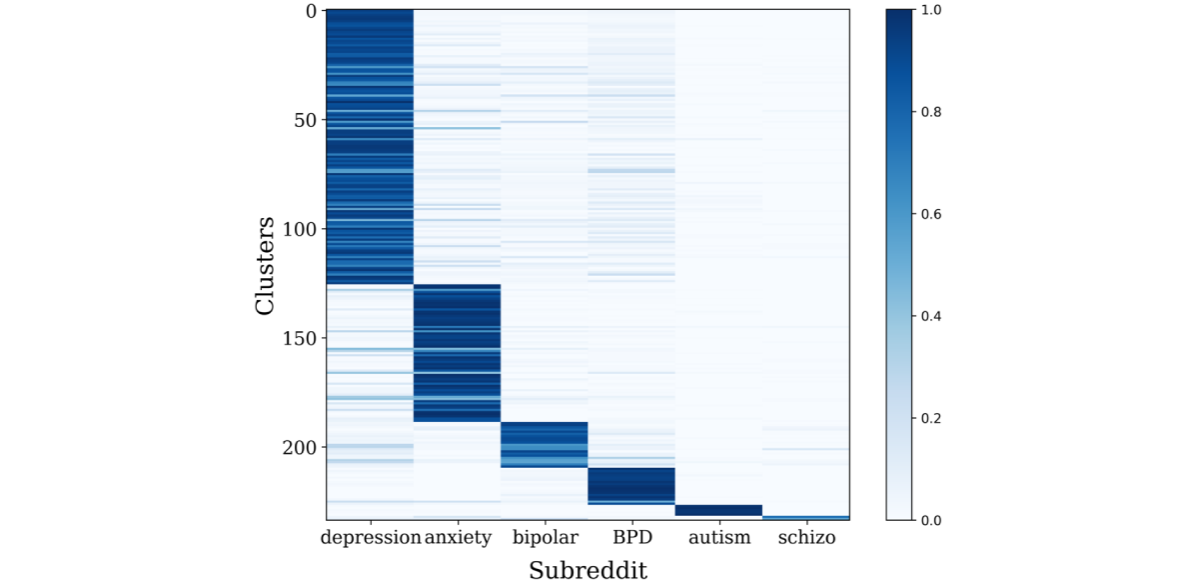
Results of the sentence-BERT internal method. BERT: bidirectional encoder representations from transformers; BPD: borderline personality disorder.

**Figure 6:**
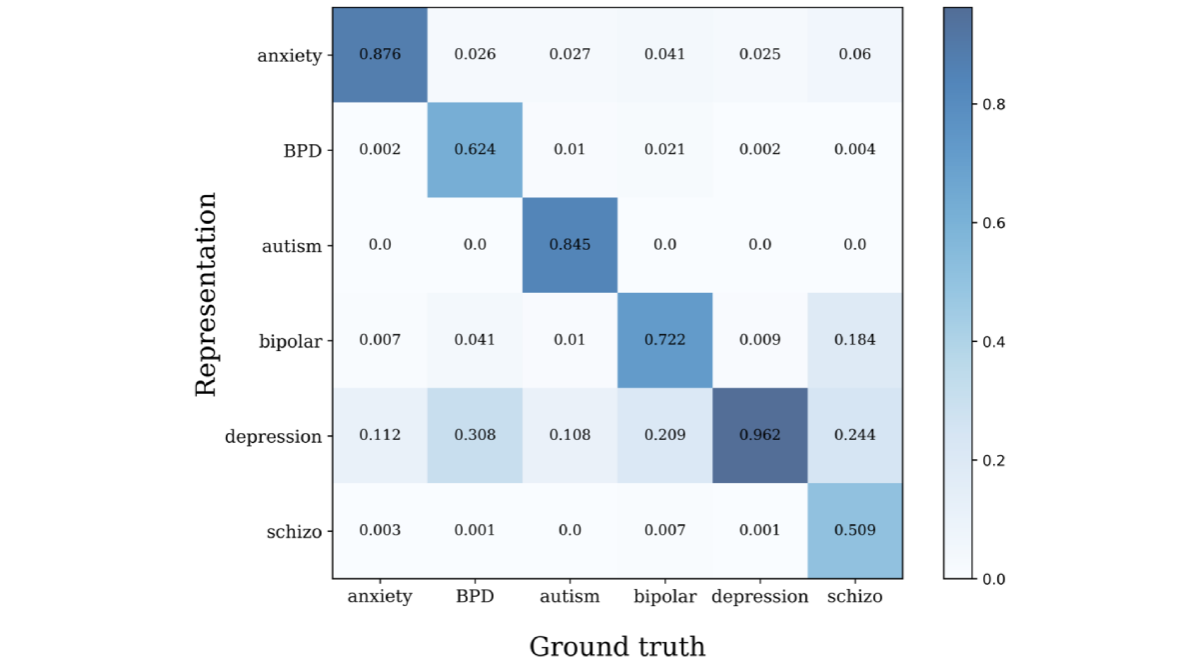
Confusion matrix for all subreddits. BPD: borderline personality disorder.

**Figure 7:**
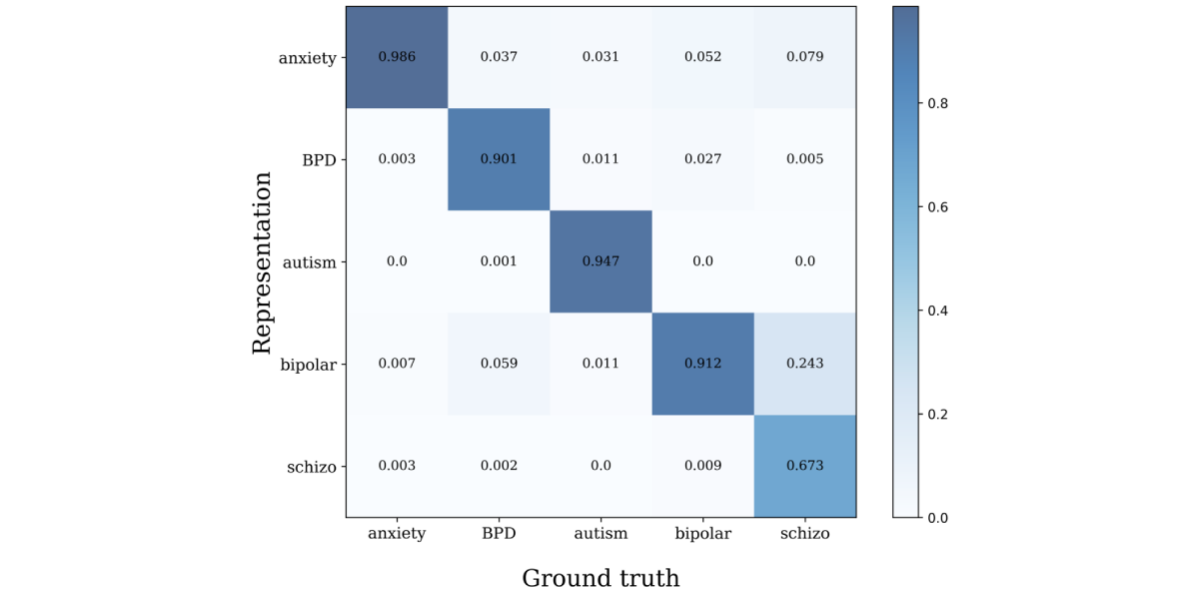
Confusion matrix excluding r/depression. BPD: borderline personality disorder.

Through sentence-BERT clustering, we observed the subreddit distribution of clusters to check whether each cluster properly represents the corresponding subreddit. In total, 234 small, fine-grained clusters were determined, and each cluster was categorized into 1 of the 6 subreddit groups, as shown in Figure 5. For a quantitative measure, we calculated the classification performance, as shown in Figure 6, with the following accuracy levels: anxiety, 87.6%; BPD, 62.4%; autism, 84.5%; bipolar, 72.2%; depression, 96.2%; and schizophrenia, 50.9%. The r/depression subreddit tended to overlap with other subreddits, which was also identified in previous studies [32-34]. Therefore, we subsequently examined the clustering performance without the r/depression subreddit. The results showed that all subreddits had higher distinctiveness, except r/schizophrenia, which had a significantly small amount of data compared to the others (anxiety, 98.6%; BPD, 90.1%; autism, 94.7%; bipolar, 91.2%; schizophrenia, 67.3%; Figure 7).

**Figure 8:**
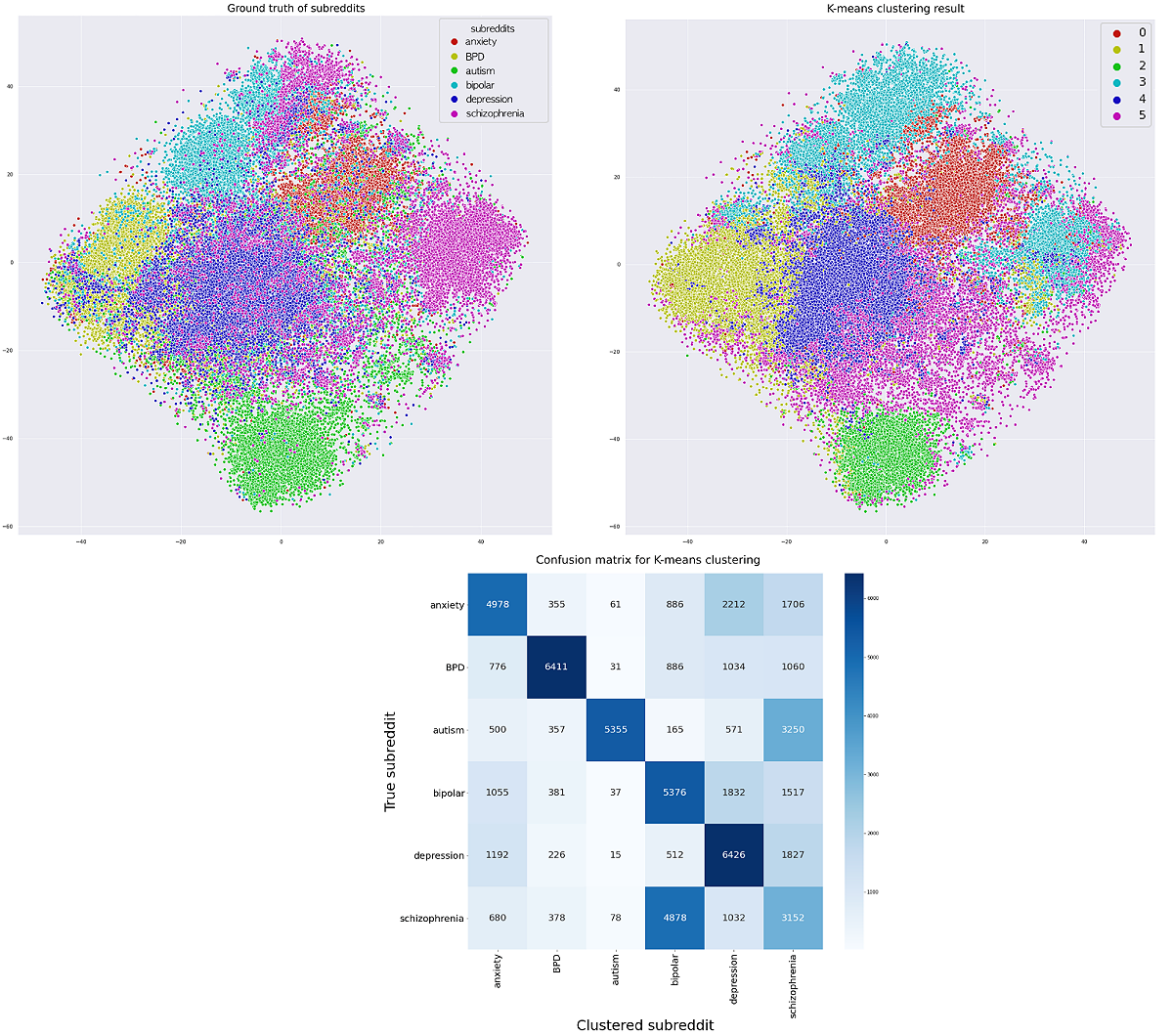
K-means clustering results and comparison with the ground truth. BPD: borderline personality disorder.

Unsupervised clustering using the k-means function after random undersampling showed adequate clustering performance. After clustering BERT embeddings into 6 clusters, for visualization, the data points were mapped into 2D space using t-SNE. We found that clustered embedding features showed similar distributive patterns as the ground truth without any supervision, exhibiting an overall accuracy of 52% (anxiety, 62.9%; BPD, 48.8%; autism, 52.5%; bipolar, 52.7%; depression, 63.0%; schizophrenia, 30.9%), as shown in Figure 8.

**Figure 9:**
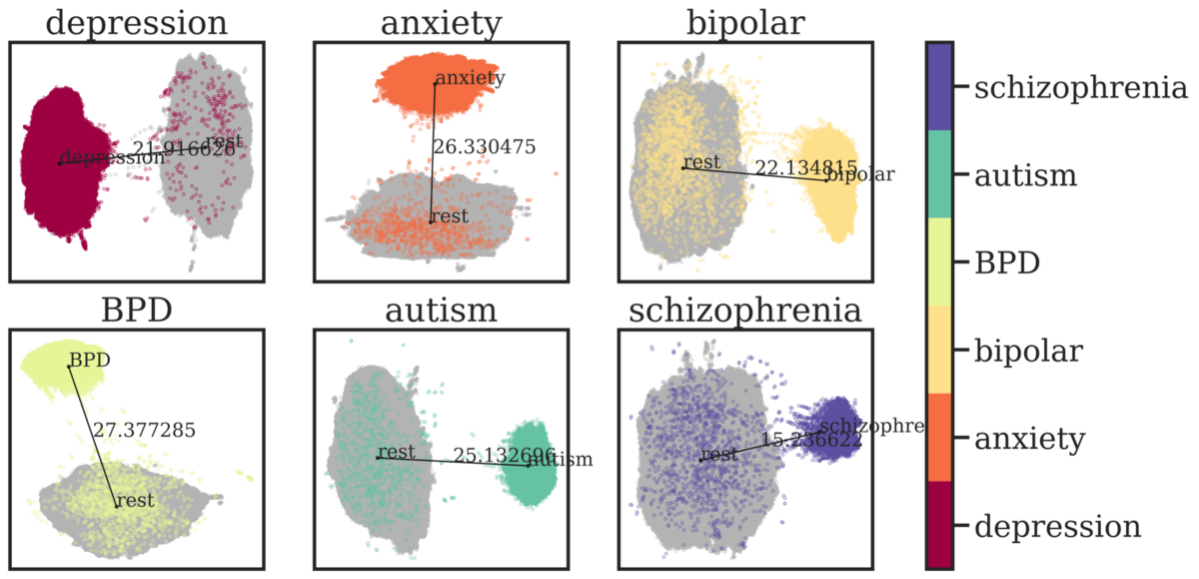
One-vs-rest supervised clustering result. BPD: borderline personality disorder.

**Figure 10:**
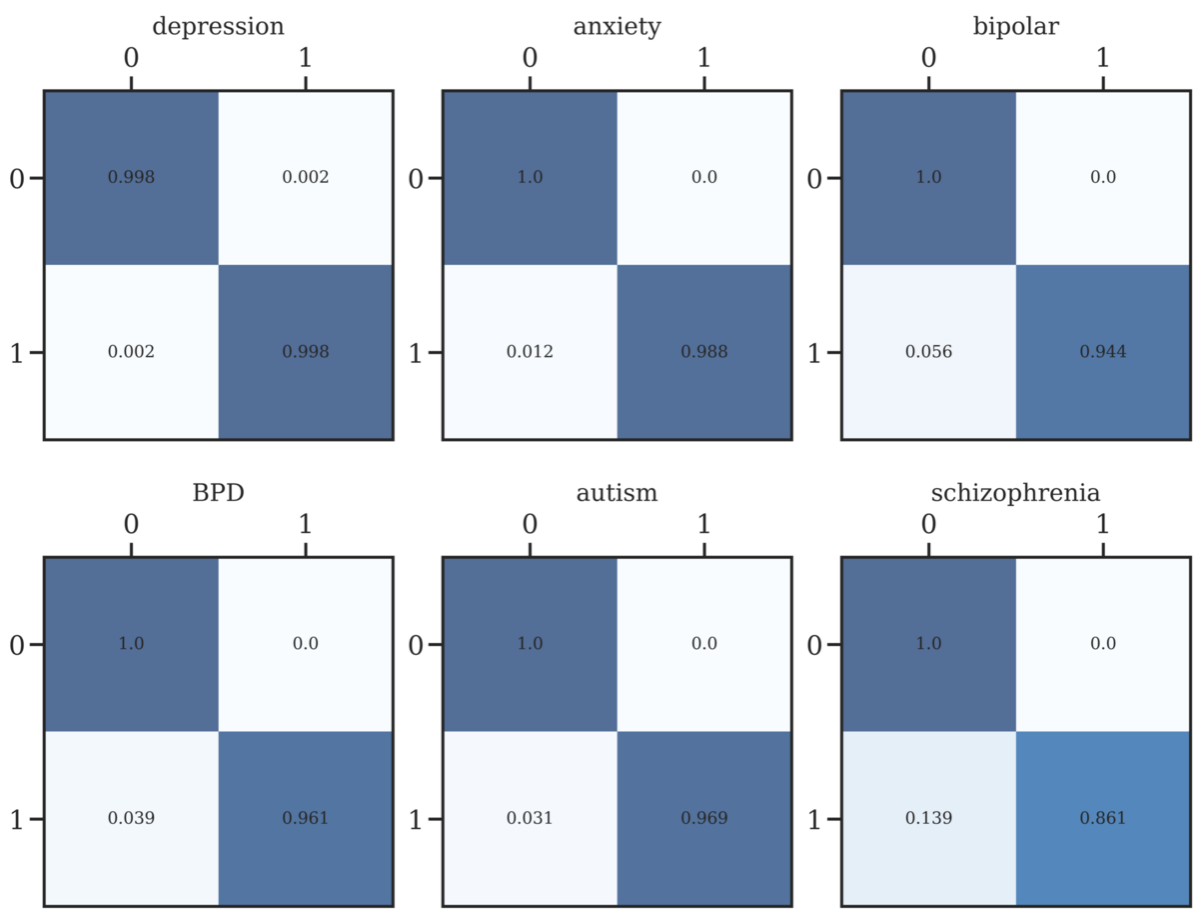
Confusion ratio for 1-vs-rest clustering. BPD: borderline personality disorder.

We applied supervised binary UMAP for all subreddits by determining that a post’s label was positive if and only if it belonged to each subreddit. The left-hand side of Figure 9 shows that each subreddit exhibited sufficient distinctiveness to be represented as different clusters in a supervised environment. We also used the HDBSCAN algorithm to quantify the effectiveness of such classification. As seen on the right-hand side of Figure 10, such approaches showed high performance in classifying embeddings into 2 clusters, ranging from 86.4% to 99.8% on each subreddit. We also reported several metrics to show how our clustering approaches performed in quantitative ways: distance between the clusters’ centroids, Davies-Bouldin scores [35], and silhouette scores [36]. Results are presented in detail in Table 9.

**Table 9:** Evaluation metrics of the 1-vs-rest clustering approach.

Subreddit	Distance between centroids of 2 clusters	Davies-Bouldin score	Silhouette score
r/depression	17.9	0.397	0.726
r/anxiety	25.5	0.263	0.791
r/bipolar	21.4	0.292	0.742
r/BPD^a^	29.1	0.222	0.803
r/autism	24.4	0.252	0.769
r/schizophrenia	15.4	0.346	0.639

^a^BPD: borderline personality disorder.

#### External Comparison

**Figure 11:**
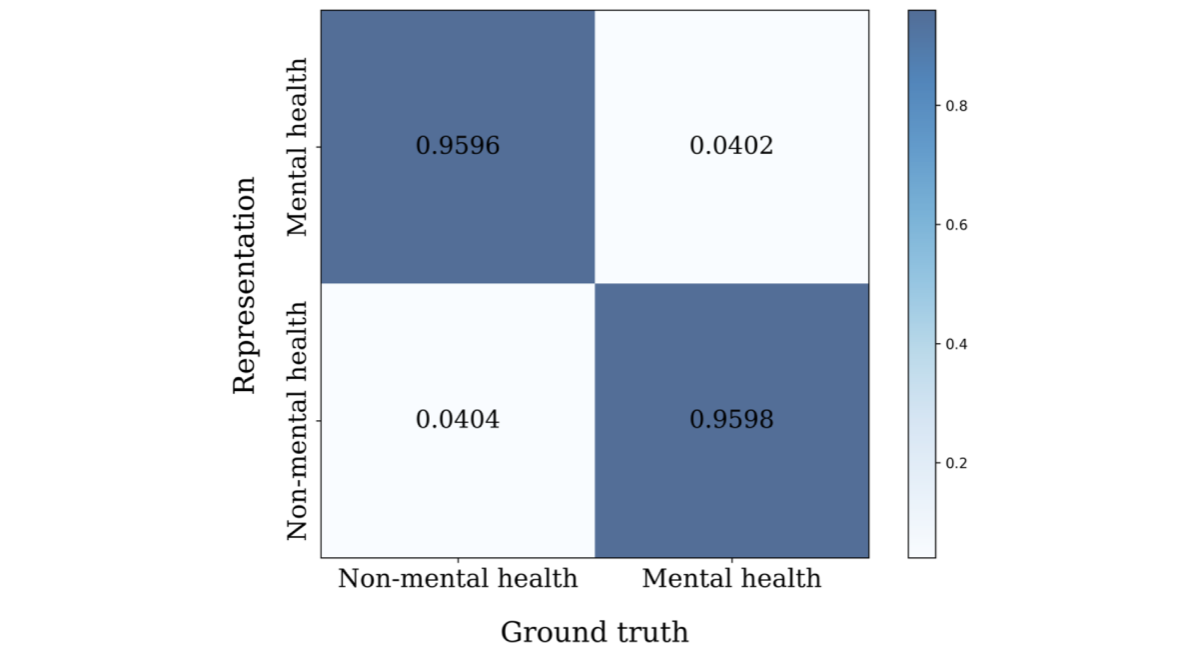
Confusion matrix of categorized sentence-BERT clusters. BERT: bidirectional encoder representations from transformers.

We observed the group distribution of clusters to check whether each cluster properly represented the corresponding group through sentence-BERT clustering. In total, 535 small, fine-grained clusters were determined from the union of non–mental health and mental health data. Each cluster was categorized into 1 of the 2 groups (non–mental health or mental health). For a quantitative measure, we calculated the classification performance, as shown in Figure 11, with the following accuracy levels: 96.0% for non–mental health and 96.0% for mental-health. The results showed that the posts of users with mental illness and users without mental illness were highly distinct from one another.

**Figure 12:**
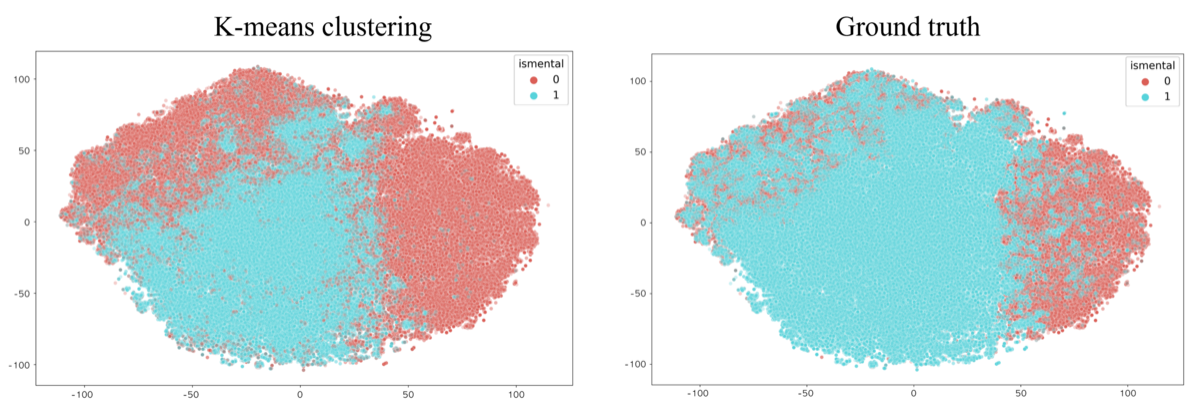
K-means clustering results and visualization compared with ground truth.

**Figure 13:**
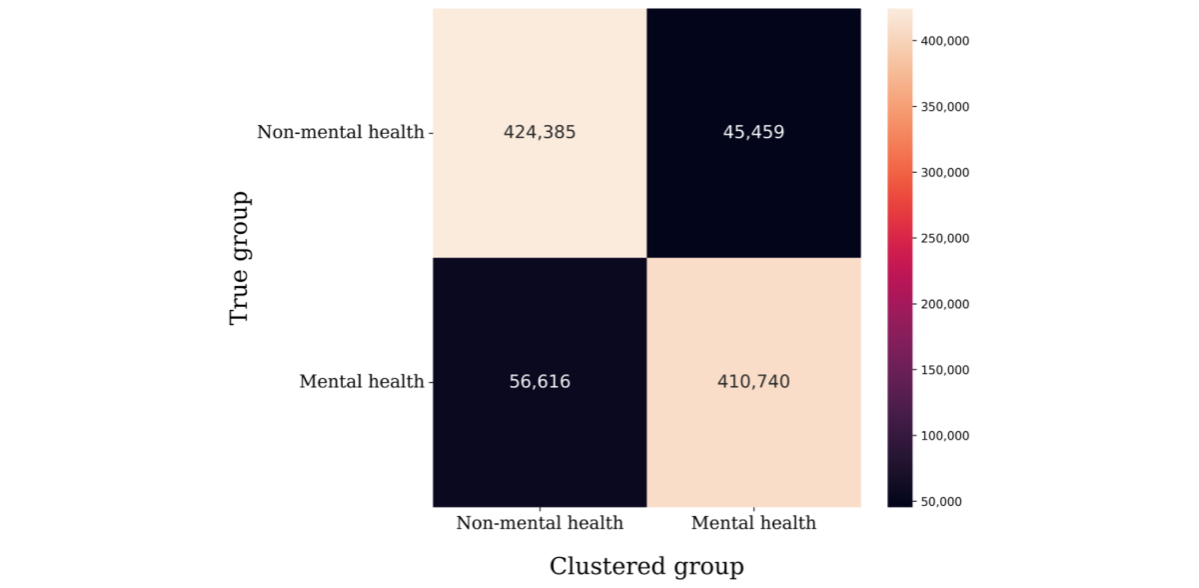
K-means clustering results and comparison with ground truth.

Unsupervised clustering using the k-means function also showed high clustering performance in the external comparison experiment. After clustering BERT embeddings into 2 clusters, t-SNE was used to visualize the data points into a 2D space. We found that the clustered embedding features showed similar distributive patterns as the ground truth without any supervision, as shown in Figure 12. Compared to the ground truth, the clustered labels exhibited an overall accuracy of 89.1%, as shown in Figure 13.

**Figure 14:**
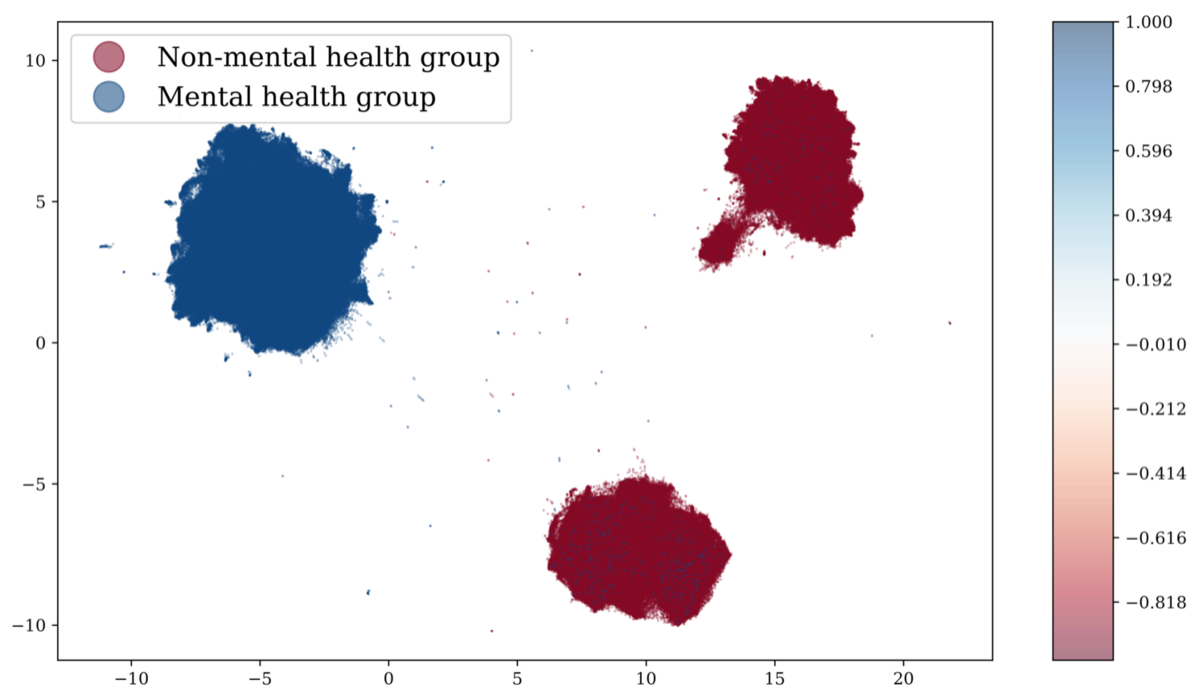
Non–mental health vs mental health supervised clustering result. UMAP: uniform manifold approximation and projection.

**Figure 15:**
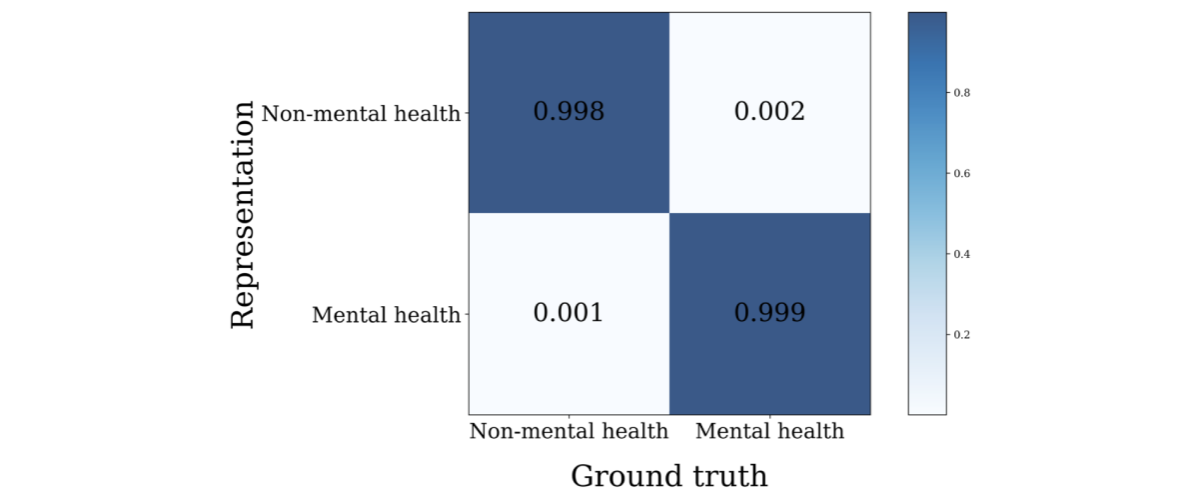
Non–mental health vs mental health binary clustering results and comparison with the ground truth. HDBSCAN: hierarchical density-based spatial clustering of applications with noise.

Finally, supervised binary UMAP was applied to determine the 2 groups of text embedding vectors. We used 30 for the number of neighbors and a minimal distance of 0.25, which was derived from a grid search. Figure 14 shows that the result exhibited sufficient distinctiveness to be represented as different clusters in a supervised environment. We also used the HDBSCAN algorithm to quantify the effectiveness of such classification. As seen in Figure 15, such approaches showed high performance in classifying embeddings into 2 clusters with classification performances of 99.8% and 99.9% for non–mental health and mental-health data, respectively. We also reported the average distance between the clusters’ centroids (21.2), the Davies-Bouldin score (1.638) [35], and the silhouette score (0.729) [36].

## Discussion

### Principal Findings

Throughout the research, we presented that people with mental illnesses, represented as mental health–related subreddit participants, show distinctive linguistic features based on their disorder. LIWC analyses and subsequent ANOVA or *t* tests revealed that people with certain mental disorders tend to have several significant linguistic features within their Reddit posts based on the single or multiple disorders they have.

Moreover, such distinctiveness is observable at the embedding level, as BERT-embedded versions of users’ posts can be efficiently classified using both supervised and unsupervised clustering algorithms. Further analyses involving a control group showed that such differences do stem from mental illnesses, as both LIWC analysis and the clustering approach showed significant differences between them. A detailed discussion of our study is stated next.

#### Linguistic Analysis of Unisubreddit Users’ Posts

The findings of this study are consistent with what has been reported in previous studies [13,37-43]. Notably, schizophrenia, recognized for causing auditory and visual disturbances [37], is reflected in users’ linguistic patterns on SNSs. Similarly, the inclination of patients with depression toward thoughts of death [38] is evident through their SNS expressions using death-related language. Moreover, since people with autism tend to show more confidence in their speech [39,40], users with autism showed a high confidence level in their linguistic presentations on SNSs. The findings also support the fact that patients with anxiety disorder have more anxious and negative thoughts than controls [41,44], and further revealed that these patterns are reflected in their SNS activity. The prevalence of ingestion-related terms in r/bipolar corresponds to previous findings associating bipolar disorder with eating disorders [42]. Meanwhile, the more frequent used of female words represents the characteristics of BPD, which is more common in women [39]. Anxiety is well known for its symptoms of expressing more negative affections and anxiety [45,46], which also supports our result.

Furthermore, the linguistic analysis conducted in this study provides nuanced insights into the terminologies used by individuals with mental health conditions on SNSs. Noteworthy is the profound usage of affiliation- and friend-related terms in the r/BPD subreddit, potentially indicative of users’ susceptibility to relationship building and concern for such issues. This aligns with prior research highlighting BPD’s features of unstable interpersonal relationships and a fear of abandonment [43].

The use of first-person singular terms (eg, I) in the r/depression subreddit might suggest that users are inclined toward higher levels of self-consciousness and shame compared to others. This interpretation finds support in numerous studies conducted within clinical settings [47-49]. The higher level of anger users with depression express on SNSs may be a consequence of symptoms of anger and anger attack, which are highly prevalent in patients with depression, as supported by precious research [50-52]. Additionally, the use of more common sexual words might suggest discussions of their experiences of sexual abuse. This speculation is rooted in the understanding that early sexual abuse can lead to depression in adulthood [53,54].

Users of the anxiety subreddit using an increased number of body-related terms could indicate a tendency to discuss on SNSs the physical abuse they encounter. This interpretation finds support in prior research that has unveiled a connection between childhood physical abuse and anxiety disorders [55-57]. It could also be linked to their heightened self-consciousness concerning body image and experiences of body shaming [58,59].

Patients with autism exhibit a greater tendency to express positive emotions in their discourse, according to our findings. This might be attributed to their communication style characterized by direct and factual language [60,61]. This manner of communication often appears emotionally neutral or positive, as it focuses on conveying information rather than conveying intricate emotions.

Finally, the relatively elevated level of nonfluency observed in the r/bipolar subreddit could stem from the cognitive impairments associated with bipolar disorder. These impairments can affect attention, concentration, and memory [62,63], which, in turn, may contribute to challenges in organizing thoughts and using fluent language.

Furthermore, as shown by the subsequent feature extraction and clustering procedures performed in this study, our data set effectively reflects each mental disorder’s characteristics, even at the feature embedding level. By using unsupervised clustering methods, each subreddit post embedding can be successfully categorized into 1 of the 6 subreddit groups, showing a strong tendency to belong to a unique subreddit and exhibiting similar distributive patterns as the ground truth. The findings also demonstrate that the majority of posts are accurately classified into their respective subreddits at both macro and micro levels. This highlights that the model can effectively capture the distinctive features of each subreddit across the 6 mental health groups using unsupervised learning alone. We also revealed that each subreddit shows sufficient distinctiveness to be represented as different clusters in a supervised environment.

#### Linguistic Analysis of Multisubreddit Users’ Posts

In addition to analyzing the characteristic differences between groups, our study confirmed that users active in 2 or more mental disorder subreddits revealed differences in language expression in each group, indicating that even the same person can reveal different tendencies in different communities on SNSs in terms of mental illness. The variations in language expression among individuals engaged in multiple mental disorder subreddits stem from diverse factors based on which individuals categorize themselves and others into social groups, leading to the adoption of group-specific behaviors and language, as supported by social identity theory [64]. Furthermore, according to social coping theory [65], which highlights how individuals adapt their coping strategies to the social context, different online mental health communities may provide varying forms of support, which users reflect through language that resonates with each community’s coping mechanisms. Thus, the identification of different language tendencies within the same individuals across mental disorder subreddits highlights the complex interplay between online community dynamics and mental health expression, providing valuable insights for both research and practice.

### External Validation Through Comparison With the Control Group

We further analyzed this distinctiveness not only internally but also externally by comparing mental health–related posts and general posts. This revealed that posts of users with mental illness can be distinguished from those of unaffected users, recognizing the disordered ways of communicating on SNSs via both linguistic and machine learning approaches. The result showed that a number of linguistic features have prominent differences, along with significantly large effect sizes (Cohen f>0.5). The overall mental health group showed relatively high usage of terms related to sadness and health. A high level of authenticity and self-consciousness words (eg, I) as well as negative emotion and affective terminologies was detected. These results are generally in line with previous studies that have compared the mentally affected group with the control group [3,28] and further open up theoretical insights for clinicians and researchers.

Our approach can also contribute to the field of recent mental disorder analysis using machine learning and deep learning through textual data and natural language processing (NLP) techniques. In addition to dictionary-based linguistic analysis, we demonstrated that text from SNSs can be effectively distinguished into 2 groups using both supervised and unsupervised clustering. This revealed that in addition to the linguistic features distinguished by LIWC, the overall semantics and textual information can be distinguishable at the level of embedded vectors through machine learning even more conveniently and effectively.

Considering this, patients’ SNS data can also be used as a useful auxiliary diagnostic means by which psychiatrists can better understand patients’ mental state and potentially make a more accurate diagnosis. Clinicians could potentially use similar approaches to identify individuals at risk or in need of intervention, even before they seek formal medical help. Early detection can lead to timely and appropriate support, improving patient outcomes and offering cost-effective mental health analysis compared to clinical questionnaires or experiments.

### Limitations

Despite the contributions of this work, as described before, this study has several limitations. First, it cannot verify that each subreddit user has been diagnosed with the disease associated with the Reddit subreddit in which they are posting. Furthermore, in the future, external validation, a process involving the verification of data by clinical experts, can be pursued.

In addition, because most Reddit users reside in English-speaking countries, only posts in English were considered in this study. Several research groups have suggested that a wider range of opinions and perspectives about the experiences of individuals from diverse geographical locations [66] can be obtained when individuals write feedback reviews in their native language. Therefore, it is considered that future studies will be able to achieve further progress by considering these limitations.

### Conclusion

As the number of people with mental illnesses increases, identifying the characteristics of those with these conditions or detecting their early signs is becoming a focus among mental health researchers. Our study aimed to analyze the linguistic characteristics of users of mental illness subreddits on Reddit, both internally and externally. Additionally, it sought to determine whether machine learning techniques can effectively classify these user groups using their SNS posts. To achieve our goal, we used LIWC to conduct linguistic analysis of SNS posts of various users. We analyzed the key linguistic features through comparisons among mental health–related subreddits (ANOVA), compared them externally with control groups (*t* test), and implemented clustering methods to evaluate whether machines can grasp and distinguish the differences. By collecting and analyzing large-scale data, this study revealed that the linguistic characteristics of those with mental diseases can be identified using data on the posts of users in each mental illness subreddit on Reddit. This study sheds light on the potential for identifying factors associated with users’ mental issues.

Overall, we contributed to the advancement of the field of mental health and text analysis research by providing a large-scale data set related to mental illness. Other researchers can use this data set to gain insights into the topic and develop new findings. In other words, the study provides a valuable resource for future investigations. Furthermore, it provides an opportunity to capture minor symptoms in advance for those unaware of their mental health status. In addition, psychiatric service providers can potentially introduce more appropriate treatments by analyzing patients’ language usage patterns or written records.

## Appendix

Multimedia Appendix 1 Supplementary materials.

## Abbreviations

BERT: bidirectional encoder representations from transformers
BPD: borderline personality disorder
HDBSCAN: hierarchical density-based spatial clustering of applications with noise
LIWC: Linguistic Inquiry and Word Count
RQ: research question
SNS: social networking service
t-SNE: t-distributed stochastic neighbor embedding
UMAP: uniform manifold approximation and projection

## Data Availability

A part of the data set is publicly available [67]. The full data set can be available if a request is submitted [68].
